# MiRNAs from serum-derived extracellular vesicles as biomarkers for uveal melanoma progression

**DOI:** 10.3389/fcell.2022.1008901

**Published:** 2022-12-22

**Authors:** Joanna Patrycja Wróblewska, Michał Stefan Lach, Marcin Rucinski, Igor Piotrowski, Lukasz Galus, Wiktoria Maria Suchorska, Stephanie Kreis, Andrzej Marszałek

**Affiliations:** ^1^ Department of Oncologic Pathology and Prophylaxis, Poznan University of Medical Sciences, Poznan, Poland; ^2^ Department of Tumor Pathology, Greater Poland Cancer Centre, Poznan, Poland; ^3^ Department of Life Sciences and Medicine, University of Luxembourg, Belval, Luxembourg; ^4^ Department of Orthopedics and Traumatology, Poznan University of Medical Sciences, Poznan, Poland; ^5^ Radiobiology Lab, Department of Medical Physics, Greater Poland Cancer, Poznan, Poland; ^6^ Department of Electroradiology, Poznan University of Medical Sciences, Poznan, Poland; ^7^ Department of Histology and Embryology, Poznan University of Medical Sciences, Poznan, Poland; ^8^ Department of Medical and Experimental Oncology, Heliodor Swiecicki University Hospital, Poznan University of Medical Sciences, Poznan, Poland

**Keywords:** uveal melanoma, extracellular vesicles, miRNA, microRNA, biomarkers, liquid biopsy

## Abstract

Uveal melanoma (UM) is a rare type of malignancy that originates from melanocytes in the choroid, iris and the eye’s ciliary body. Biomarkers for early detection and progression of UM, especially the molecular traits governing the development of metastasis, are still not available in clinical practice. One extensively studied components of liquid biopsies are extracellular vesicles. Due to their unique molecular cargo, they can contribute to early cancer development and at the same time carry markers for disease onset and progression. For characterisation of the miRNA profiles present in circulating serum-derived exosomes of patients with diagnosed primary and metastatic UM, we have analyzed the miRNA cargos using next-generation sequencing followed by RT-qPCR validation in a cohort of patients (control n = 20; primary n = 9; metastatic n = 11). Nine miRNAs differentiating these patient groups have been established. We show that hsa-miR-144-5p and hsa-miR-191-5p are the most promising biomarker candidates, allowing the categorization of patients into local and advanced UM. Additionally, the comparison of miRNA expression levels in exosomes derived from UM patients with those derived from healthy donors revealed that hsa-miR-191-5p, -223-3p, -483-5p, -203a has the potential to be used as an early marker for the presence of UM. This pilot study reveals that miRNAs extracted from circulating exosomes could be exploited as potential biomarkers in UM diagnosis and, more importantly, for indicating metastatic spread.

## 1 Introduction

Uveal melanoma (UM) is one of the most aggressive cancers and the most common primary intraocular malignancy in adults arising from melanocytes localized in the choroid (approximately 90% of cases), ciliary body (∼5%) and iris (∼3%) (Berus et al., 2017; Krantz et al., 2017). Although relatively rare, as it accounts for less than 5% of all melanoma cases, it is associated with high mortality, exemplified by a 2-year survival rate of only 8% (Kaliki and Shields, 2017). Primary UM tumors are relatively well managed with either brachytherapy for small, localized tumors or surgery followed by radio- or chemotherapy for tumors with sizes above 1,5 cm or invading the optic nerve. However nearly half of the UM patients will progress to metastatic disease (Damato, 2018). For metastatic stages of UM, systemic therapies, including chemo- and immunotherapy, are currently administered, albeit with limited efficacy, leaving early detection and management of primary tumors with surgery and/or chemo-/radiotherapy as the most successful treatment option (Damato, 2018).

The risk for developing metastases reaches over 50%, with the liver being the most common metastatic site. Histological and genetic features of UM tumors related to poor prognosis and increased risk of developing metastatic disease have been described. They include tumor size, chromosome 3 monosomy, loss of BRCA1 associated protein-1 (*BAP1*), eukaryotic translation initiation factor 1 (*EIFAX*) and splicing factor 3 subunit 1 (*SF3B1*) mutations (increasing the risk of metastases in disomy 3 tumors) (Kaliki and Shields, 2017; Robertson et al., 2017).

Early detection of uveal melanoma progression into metastatic disease remains challenging since there are no reliable, easy-to-use molecular biomarkers. The diagnosis of patients is usually delayed due to the “hidden” nature of the intraocular tumors and non-specific symptoms. Several studies have focused on discovering liquid biopsy biomarkers to identify UM risk factors responsible for metastatic spread (Madic et al., 2012; Bidard et al., 2014; Stark et al., 2019; Jin and Burnier, 2021). Over the past decade, liquid biopsies have been receiving increasing attention, primarily with the analysis of circulating tumor cells (CTC), followed by circulating tumor DNA (ctDNA) as well as other particles: cell-free RNA, extracellular vesicles, cancer-related proteins and metabolites (Alix-Panabières and Pantel, 2021). Liquid biopsies hold a great potential in improving cancer diagnostics, combining sensitivity, specificity, ease of implementation and minimal invasiveness for the patient. Furthermore, this type of clinical sample allows for evaluating disease progression, response to treatment and risk of recurrence. Currently, in addition to the FDA-approved CELLSEARCH platform for CTC, qPCR-based *EGFR*, *KRAS* and *PIK3CA* mutation detection tests and NGS-based gene panel analysis, further attention is paid to extracellular vesicles and their cargo in the context of liquid biopsies (Alba-Bernal et al., 2020; Ignatiadis et al., 2021).

Among the potential sources for liquid biopsy purposes, a family of extracellular vesicles (EVs) recently have gained much interest due to their unique properties. Amongst them, exosomes are the smallest and the most intensively studied group, representing bilayer-lipid blebs below 150–200 nm in diameter. They contain biomolecules such as proteins, lipids and nucleic acids, characteristic of their cells of origin (Cesi et al., 2016). Although all cells studied so far produce exosomes, malignant cells are known to produce a higher quantity of tumor-derived exosomes, (TEX) and the ratio of TEX to healthy cell-derived exosomes in cancer patient’s blood is higher in patients with advanced disease, high tumor burden and metastatic disease (Ludwig et al., 2019; Walbrecq et al., 2020). Apart from shaping the tumor microenvironment to allow for distant metastasis, exosomes also influence the development of drug resistance and escape of tumor cells from immune surveillance (Costa-Silva et al., 2015; Lobb et al., 2017; Barok et al., 2018).

Previously, we have reported potential protein biomarkers, derived from UM extracellular vesicles that allow distinction between healthy individuals and uveal melanoma patients with either primary or metastatic disease. We also showed the possibility of using these markers to detect the metastatic spread of UM (Wróblewska et al., 2021).

Recent studies suggest that exosomes transport most cell-free miRNAs as molecular cargo (Bryzgunova et al., 2021). Moreover, miRNA profiles were analyzed in exosomes derived from liver perfusates of vitreous humor from metastatic UM patients and isolated from serum samples of UM patients with primary tumors only (Eldh et al., 2014; Ragusa et al., 2015). However, none of these studies addressed the differences in miRNA levels between exosomes derived from patients at different disease stages.

Here, we analyzed the miRNA cargo of exosomes derived from serum samples of uveal melanoma patients diagnosed with either primary or metastatic disease and healthy donors. The aim of the study was to identify potential biomarkers for simple and minimally invasive screening allowing for early detection of UM tumor development and to assess the risk of metastatic spread.

## 2 Materials and methods

### 2.1 Study group

The study was approved by the Bioethics Committee of Poznan University of Medical Sciences (number 114/18). Twenty UM patients with either primary or metastatic disease and 20 healthy donors were enrolled. The inclusion criteria for patients included: UM of the choroid, confirmed by histopathological assessment of tumor tissue, tumor size> 1 cm, presence of liver metastasis (for the metastatic group only), no previous cancer, no prior oncologic treatment, no immunological diseases. The blood samples were collected before the implementation of any therapy (surgery in case of primary patients or systemic therapies in case of metastatic patients).

The healthy donor’s group included healthy individuals, females and males, age 30–60, matching the time of UM onset. The exclusion criteria for donors were: previous or ongoing immunological disease or cancer, ongoing viral or bacterial infection, ongoing immunosuppressive therapy, and any vaccination in the period of 3 months before blood collection.

### 2.2 Exosome isolation

Whole blood from UM patients and healthy donors was collected using S-Monovette^®^ Serum Gel tubes with clotting activator and separating gel (Sartstedt, Newton, NC, United States) and centrifuged at 2500 x g for 10 min to isolate the serum, which was aliquoted and frozen at -80 °C. Exosomes were isolated using several centrifugation steps as previously described (Wróblewska et al., 2021; Wyciszkiewicz et al., 2021). First, thawed serum was centrifuged for 30 min at 500 x g. Further, to get rid of larger debris and apoptotic bodies, the supernatant was centrifuged for 45 min at 12,000 x g. Then, it was filtered using 0.2/0.8 µm Acrodisc^®^ PF syringe filters (Pall, Port Washington, NY, United States) and transferred to Amicon^®^ Ultra-15 Centrifugal Filter Unit with a 100 kDa cut-off (Merck KGaA, Darmstadt, Germany), filled with Phosphate-Buffered Saline (PBS) (Biowest, Nuaillé, France) and centrifuged for 15 min at 5000 rpm to enrich the exosomal fraction and to decrease the viscosity of the sample. Concentrated samples were diluted again in PBS, transferred to ultracentrifuge tubes and centrifuged for 90 min at 120,000 x g (fixed angle rotor 70.1 Ti, Beckman Coulter, Indianapolis, IN, United States). All centrifuge steps were performed at 4°C. Obtained pellets were resuspended in RIPA Lysis buffer containing protease inhibitor cocktail (both obtained from Sigma-Aldrich, St. Louis, MO, United States) or PBS depending on the further assay. The concentration of exosomal proteins (for Western blot analysis) was determined using the Pierce™ BCA Protein Assay Kit (ThermoFisher Scientific, Waltham, MA, United States), aliquoted and frozen at -80°C for further analysis. The concentration of exosomes was analyzed using Nanoparticle Tracking Analysis (NTA).

### 2.3 Western blot analysis of exosomes

Western blot analysis was performed using 20 µg of protein per lane to confirm the successful isolation of exosomes. The sample was mixed with 4x Laemmli buffer (Bio-Rad, Hercules, CA, United States) and heated for 10 min at 95°C. Then, cooled samples were transferred to 4–20% gradient polyacrylamide gel Stain-Free Mini-PROTEAN^®^ TGX™ Precast Protein Gels (Bio-Rad, Hercules, CA, United States). After electrophoresis, the gel was activated using ChemiDoc™ Touch Imaging System (Bio-Rad Laboratories, Hercules, CA, United States) in order to detect the proteins after their transfer to a polyvinylidene fluoride (PVDF) membrane (Bio-Rad, Hercules, CA, United States). The membrane was blocked in 5% non-fat dry milk (Sigma Aldrich, St.Louis, MO, United States) prepared in Tris-buffered saline containing 0.1% Tween 20 (TBST) (Tris Base, Sodium chloride both obtained VWR Chemicals, Leuven, Belgium; Tween 20, POCh, Gliwice, Poland) for 2 h at room temperature. Then, the membrane was incubated overnight at 4°C with primary antibody against proteins related to exosomal origin – LAMP1, CD63 (both 1:500, Santa Cruz, Dallas, TX, United States); functional marker TGFβ and IL6 (both 1:1000, Cell Signaling Technology, Leiden, Netherlands) and a negative marker of exosomes – calnexin (1:500, Santa Cruz, Dallas, TX, United States. The next day, membranes were washed with TBST and underwent a 2-hour incubation at room temperature with a secondary antibody conjugated with horseradish peroxidase (HRP) (1:2000, Cell Signaling Technology, Leiden, Netherlands) diluted in 5% non-fat dry milk in TBST. In order to detect the proteins of interest, a chemiluminescent reaction was performed using Clarity Western ECL Substrate (Bio-Rad, Hercules, CA, United States). The membranes were documented using ChemiDoc™ Touch Imaging System. The protein bands were quantified using Image Lab Software v6.1.0 (Bio-Rad, Hercules, CA, United States). As a control, whole-cell lysates of the cutaneous melanoma cell line WM-266-4 was used.

### 2.4 Cryo-Electron microscopy

The EVs for cryoEM were isolated from pooled serum samples from healthy donors, primary UM patients and metastatic UM patients, resulting in 3 separate EV samples. The size and shape of extracellular vesicles were analyzed with cryogenic transmission electron microscopy (Cryo-EM). Briefly, 10 µL of each EV sample were snap frozen onto glow discharged 200 mesh copper microscopy grids (Jena Bioscience, Jena, Germany) using an automatic plunge freezer EM GP2 (Leica Microsystems, Wetzlar, Germany) with 30s pre-blotting incubation and 4s blotting. The images were taken using JEOL JEM 2100-Plus 200 kV transmission electron microscope (JEOL, Akishima, Tokyo, Japan) with 200 kV acceleration voltage protocol under 4×10^5^ magnification. Around 20 pictures were taken for each sample and the shape and size of obtained EVs were analyzed in all of them.

### 2.5 Nanoparticle tracking analysis

The size and concentration of isolated extracellular vesicles were analyzed by Nanoparticle Tracking Analysis (NTA) using the NanoSight NS300 instrument (Malvern Panalytical, Malvern, Great Britain). Samples were diluted with sterile PBS to obtain the concentration of 20–60 particles per frame. Three replicate video recordings of 60-s duration per sample were collected. Statistical analysis and graph plotting of resulted data was performed with the NanoSight NTA software version 3.2.

### 2.6 Exosome staining

Exosomes were stained with PKH67 Green Fluorescent Cell Linker Mini Kit for General Cell Membrane Labeling (Sigma Aldrich, St. Louis, MO, United States). Briefly, 100 µg of exosomes was diluted to a volume of 100 µL with Diluent C, mixed with 3 µL of PKH67 dye and incubated at room temperature for 5 min. Next, exosomes were transferred to Exosome Spin Columns (MW 3000) (Invitrogen, Carlsbad, CA, United States) and centrifuged 750 *g* for 2 min to remove unbound dye. The final concentration of exosomal protein was adjusted to 1 μg/μL.

### 2.7 Isolation of peripheral blood mononuclear cells

The blood of four healthy donors enrolled in the study was used as a source of peripheral blood mononuclear cells (PBMC). Written consent was obtained from all subjects. 5 ml of blood was collected from each donor in EDTA-coated tubes (Sarstedt, Numbrecht, Germany), and PBMCs were isolated immediately after collection. In order to obtain buffy coat containing a sufficient quantity of PBMCs for the planned experiment, equal volumes of blood from each donor were pooled. Pooled blood samples were diluted with PBS (1:1 v/v), and slowly overlaid on 15 ml of room temperature Ficoll Paque Plus solution (GE17-1440–02, Sigma-Aldrich, St. Louis, MI, United States). The samples were centrifuged at 400 x g for 30 min at room temperature without brakes. PBMCs were carefully collected from the interphase fraction, washed with PBS, and suspended in culture medium RPMI 1640 (Biowest, Neauville France) supplemented with 1% penicillin/streptomycin 10,000 U/mL (Biowest, Neauville, France) and 10% exosome-depleted fetal bovine serum (commercially available exosome-depleted FBS, cat no. A2720801, ThermoFisher, ThermoFisher Scientific, Waltham, MA, United States), inactivated at 56 °C for 30 min and sterile filtered through 0.22 µm filter. To measure the concentration of PBMCs before plating, we used cytometric analysis with CytoFLEX (Beckman Coulter, Pasadena, CA, United States) of samples based on forward scatter and side scatter intensity.

### 2.8 Exosome uptake assay

EA.hy926 (ATCC, Manassas, VA, United States) endothelial cells and MRC-5 pd19 lung fibroblasts (ECACC, Salisbury, United Kingdom) were both cultured in (DMEM) containing 10% fetal bovine serum (10% FBS) and 4 mM L-glutamine, 1% penicillin/streptomycin 10,000 U/mL (all provided from Biowest, Neauville, France) and 1% non-essential amino acids (Sigma Aldrich, St. Louis, United States). Between the 2^nd^ and 5^th^ passage, cells were used to perform exosomes uptake assays. Before exposition, the cells were seeded onto a 12-well plate (1 × 10^5^ cells/well) and left overnight for attachment. The next day, cells were washed twice using PBS, and fresh medium containing exosomes-depleted FBS was used (commercially available exosome-depleted FBS, cat no. A2720801, ThermoFisher Scientific, Waltham, MA, United States). The PBMC, Ea.hy926 and MRC-5 pd19 was incubated with 10 μg/ml of PHK67 labelled exosomes for 4 h in 1 ml of the medium. After incubation, cells were washed with PBS and incubated for 3 min with 1x Trypsin-EDTA solution (Sigma Aldrich, St. Louis, MI, United States) to remove non-internalized exosomes from cell membranes to avoid false-positive signals during flow cytometry analysis. Next, cells were washed with PBS and analyzed with CytoFLEX (Beckman Coulter, Pasadena, CA, United States) flow cytometer and FlowJo™ v10.6.1 software (FlowJo LCC, Ashland, OR, United States). Cells incubated with PBS/PKH67 mix, processed in a similar manner as PBS/PKH67/exosomes, were used as negative control (NTC – non-treated control).

### 2.9 MicroRNA expression profiling by next-generation sequencing

Next-generation sequencing was performed by Exicon’s Exosomes microRNA Sequencing Service (Qiagen, Hilden, Germany). The analysis was performed on exosomes samples isolated from 3 primary and 3 metastatic patients. Briefly, the miRNAs from exosomes were isolated with exoRNeasy Midi Kit (Qiagen, Hilden, Germany). The quality of obtained miRNAs was assessed by qPCR to determine if the expression levels of miRNAs were within the expected range previously described for miRNA content of biofluids (Margue et al., 2015). Hsa-miR-103a-3p, hsa-miR-191-5p, hsa-miR-451a, hsa-miR-23a-3p and hsa-miR-30c-5p were analyzed together with inhibition of enzymatic reactions (spike-in control UniSp6) and potential hemolysis (hsa-miR-23a and hsa-miR-451a). All samples passed the quality-control step and were submitted to library preparation using QIAseq miRNA Library Prep kit for the Illumina platform, according to the manufacturer’s instructions. Sequencing was performed on Illumina NextSeq500 with 12 million reads per sample. The raw data were de-multiplexed, and FASTQ files were obtained using bcl2fastq software (Illumina inc., San Diego, CA, United States). The quality of the sequencing was checked using FastQC tool. Next, the adapter sequences were trimmed from raw reads. As the length of miRNAs is approximately 18–22 nucleotides, reads of 30–50 nt length were removed. Bowtie2 was used to map the obtained reads to the miRNA database - miRBase and the human reference genome (Hg19) (Langmead and Salzberg, 2012). Differential expression study was performed using EdgeR Bioconductor package (Robinson et al., 2009). MiRNAs with *p* < 0.05 and 20% FDR correction were considered as significantly different. To identify potential target genes for differentially expressed miRNAs, the SpidermiR Bioconductor package was applied. Differentially expressed miRNAs were used as a query to search the target genes in the following databases: for predicted targets - DIANA, Miranda, PicTar, TargetScan, and for experimentally confirmed targets - miRTAR, miRwalk (Cava et al., 2017). ENTREZ IDs for the target genes were subjected to gene ontology enrichment analysis using the clusterProfiler package (Yu et al., 2012). The analysis was performed separately for each differentially expressed miRNA. Reference GO annotation data was obtained directly from the human annotation library “org.Hs.eg.db”. Enriched GO BP terms were visualized as circos plots (www.circos.ca).

### 2.10 Quantitative real-time PCR for miRNA expression

MicroRNAs were isolated from exosomes of uveal melanoma patients and healthy donors with exoRNeasy Midi Kit (Qiagen, Hilden, Germany). The cDNA was prepared from 2 µL of isolated miRNA with TaqMan™ Advanced miRNA cDNA Synthesis Kit (Applied Biosystems, Foster City, CA, United States) according to the manufacturer’s instructions.

Quantitative real-time PCR analysis was performed with TaqMan™ Advanced miRNA Assay specific to chosen miRNA and TaqMan™ Universal PCR Master Mix (Applied Biosystems, Foster City, CA, United States). All real-time based analyses were performed on a Cobas z4800 device with the LightCycler 480 Software (Roche, Basel, Switzerland). The results were displayed as relative miRNA fold change based on calculated log2-ΔΔCt values. Exogenous spike-in, hsa-miR-6351-5p mimic (Applied Biosystems, Foster City, CA, United States), was used for data normalization.

### 2.11 Statistical analysis

Statistical analyses were performed using the GraphPad Prism software program, v.9 (GraphPad Software, Inc., La Jolla, CA, United States). In order to use appropriate test, the distribution of the data was tested using the Shapiro-Wilk normality test. Data that followed Gaussian distribution were analyzed with ordinary one-way ANOVA, and those which were not normally distributed were analyzed with the Mann–Whitney test or the Kruskal–Wallis with the Dunn’s *post hoc* test. *p* < 0.05 was considered to indicate a statistically significant difference. The results were visualized as boxplots (median and whiskers) presenting relative expression levels of miRNA. Whiskers were calculated using the Tukey method based on GraphPad Prism software. *p < 0.05, **p < 0.01, ***p < 0.001. A receiver operating characteristic (ROC) curve was generated to illustrate the diagnostic usefulness of analyzed miRNA based on calculated specificity, sensitivity and area under the curve (AUC).

## 3 Results

### 3.1 Characterization of exosomes derived from serum of uveal melanoma patients

Twenty patients diagnosed with primary or metastatic UM (UM-exo primary and UM-exo metastatic) and 20 healthy donors (HD-exo) were enrolled in this study based on diagnosis (uveal melanoma localized in choroid), tumor grading, stage of the disease (primary tumor only or metastatic disease) and implemented therapy. The group further described as “primary” (n = 9) included patients diagnosed with uveal melanoma of the choroid without metastasis and before implementation of any therapy (surgery or radiotherapy) on the day of enrolment. The “metastatic” group (n = 11) consisted of patients with diagnosed UM of the choroid with confirmed metastatic spread to the liver, before implementation of systemic therapy (radio-, chemo- or immunotherapy) (Table 1). The control group was recruited from healthy donors (n = 20), with age and sex distribution matching the UM study group.

**TABLE 1:** The clinical, histopathological, and molecular characteristics of distinguished subgroups of studied primary tumor samples collected from uveal melanoma (UM) and healthy donors. na – not applicable.

Patients characteristics	Primary *n* = 9	Metastatic *n* = 11	Healthy donors n = 20
Age of diagnosis (years)			
Mean ± SD	64 ± 11	66 ± 8	50 ± 10
Gender, n			
Male	4	6	9
Female	5	5	11
Time from Pathologic Diagnosis to Follow-up or Metastasis, days Mean ± SD	365 ± 130	353 ± 260	na
AJCC Primary Tumor, *n*			
T1	0	1	na
T2	1	0	
T3	3	6	
T4	5	4	
Chromosome 3, *n*			
Monosomy	6	7	na
Disomy	3	4	
BAP1, *n*			
Positive	3	2	na
Negative	6	9	

**FIGURE 1:**
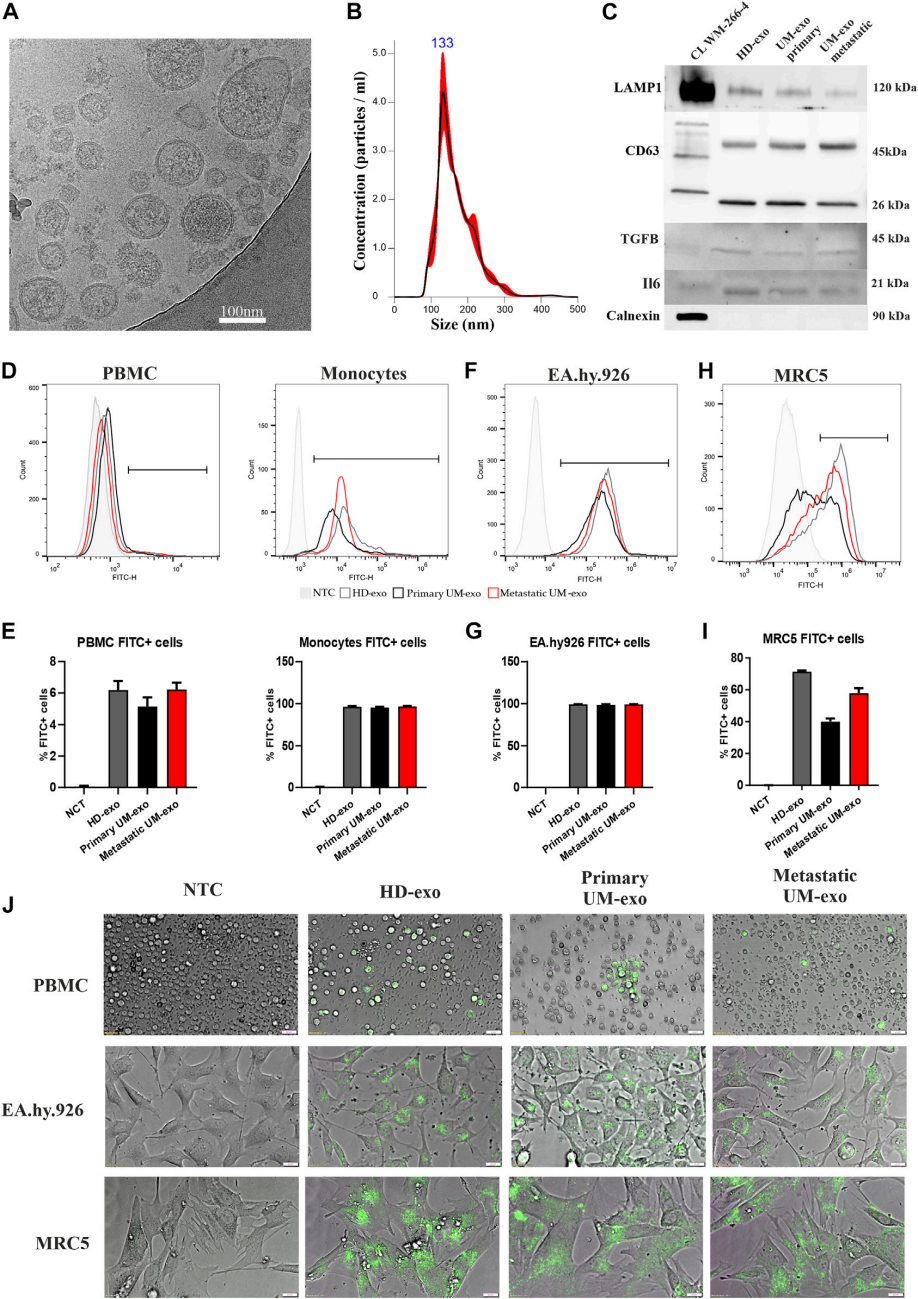
The analysis of exosomes isolated from the serum of uveal melanoma patients with primary and metastatic disease (UM-exo primary and UM-exo metastatic), and healthy donors (HD-exo). The cryogenic electron microscopy showed proper shape and structure of EVs **(A)**. The size distribution of isolated vesicles was ranging between 50 and 200 nm **(B)**. The expression of protein markers of EVs (LAMP1, CD63), their protein cargo (TGFB1, IL6 and absence of organelle-specific proteins (Calnexin) was confirmed by western blot. **(C)**. The flow cytometric analysis of uptake and internalization of fluorescently-labelled (PKH67) exosomes isolated from different patient groups (5 patients/healthy donors of each group pooled together) by PBMCs **(D**,**E)**, endothelial cells (EA.hy.926) **(F**,**G)** and lung embryonal fibroblasts (MRC5) **(H**,**I)**. Further analysis of exosomes obtained from the serum of uveal melanoma patients with primary and metastatic disease (UM-exo primary and UM-exo metastatic) and healthy donors (HD-exo) showed the ability of the PKH67-labelled exosomes (green fluorescence) to be actively taken up by PBMCs, endothelial cells and lung fibroblasts. NTC – non-treated control (PBS) **(J)**.

The extracellular vesicles were isolated from serum samples using a serial ultracentrifugation method as described previously (Wróblewska et al., 2021). Phenotypic analysis with cryogenic electron microscopy confirmed the presence of bi-layered vesicles with sizes below 200 nm (Figure 1A). Using Nanoparticles Tracking Analysis we showed the size distribution of obtained EVs to range between 50 and 200 nm (Figure 1B). Analysis of the EV’s protein cargo confirmed the expression of lysosomal associated membrane protein (LAMP) and two forms of CD63 (lower band - core protein; upper band – protein complex), which confirms the endosomal origin of isolated vesicles. TGFβ and IL6, representing functional cargo of EVs, were detected more abundantly in EVs than in this specific melanoma cell line lysate. The absence of Calnexin expression, a protein related to the endoplasmic reticulum confirmed the purity of isolated EVs (Figure 1C). Next, we tested the ability of different cell types to uptake the EVs and internalize their content. Using flow cytometry, we showed that fluorescently labelled exosomes are taken up and internalized by peripheral blood mononuclear cells (PBMC), especially by the monocytes’ subpopulation (Figure 1D), where the percentage of positive cells oscillated around 95% (Figure 1E). EA.hy.926 endothelial cells were similarly potent in taking up exosomes (Figure 1F), showing 99% of exosome-positive cells (Figure 1G). Lung fibroblasts, MRC5, showed more variability in taking up exosomes derived from different sources (Figure 1H), resulting in between 40% and 70% of positive cells (Figure 1I). Similar observations were also made by microscopy analysis of PBMCs, EA.hy.926 and MRC5 cell lines (Figure 1J).

### 3.2 The analysis of miRNA expression profiles in exosomes isolated from sera of UM patients

**FIGURE 2:**
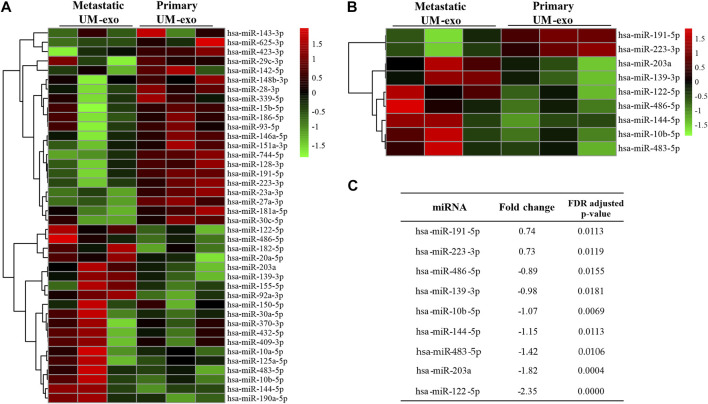
MiRNAs differentially expressed (DE) in exosomes derived from 3 patients each with primary (Primary UM-exo) or metastatic disease (Metastatic UM-exo). **(A)** Heatmap showing top 40 DE miRNAs (20 up-, 20 down-regulated) in EVs from metastatic UM patients compared to EVs from primary UM patients; **(B)** Heatmap with statistically significant DE miRNAs in EVs from metastatic *versus* primary UM patients; **(C)** Table showing log2 fold changes and FDR corrected *p*-values for differentially expressed miRNAs in EVs from metastatic *versus* UM patients.

**FIGURE 3:**
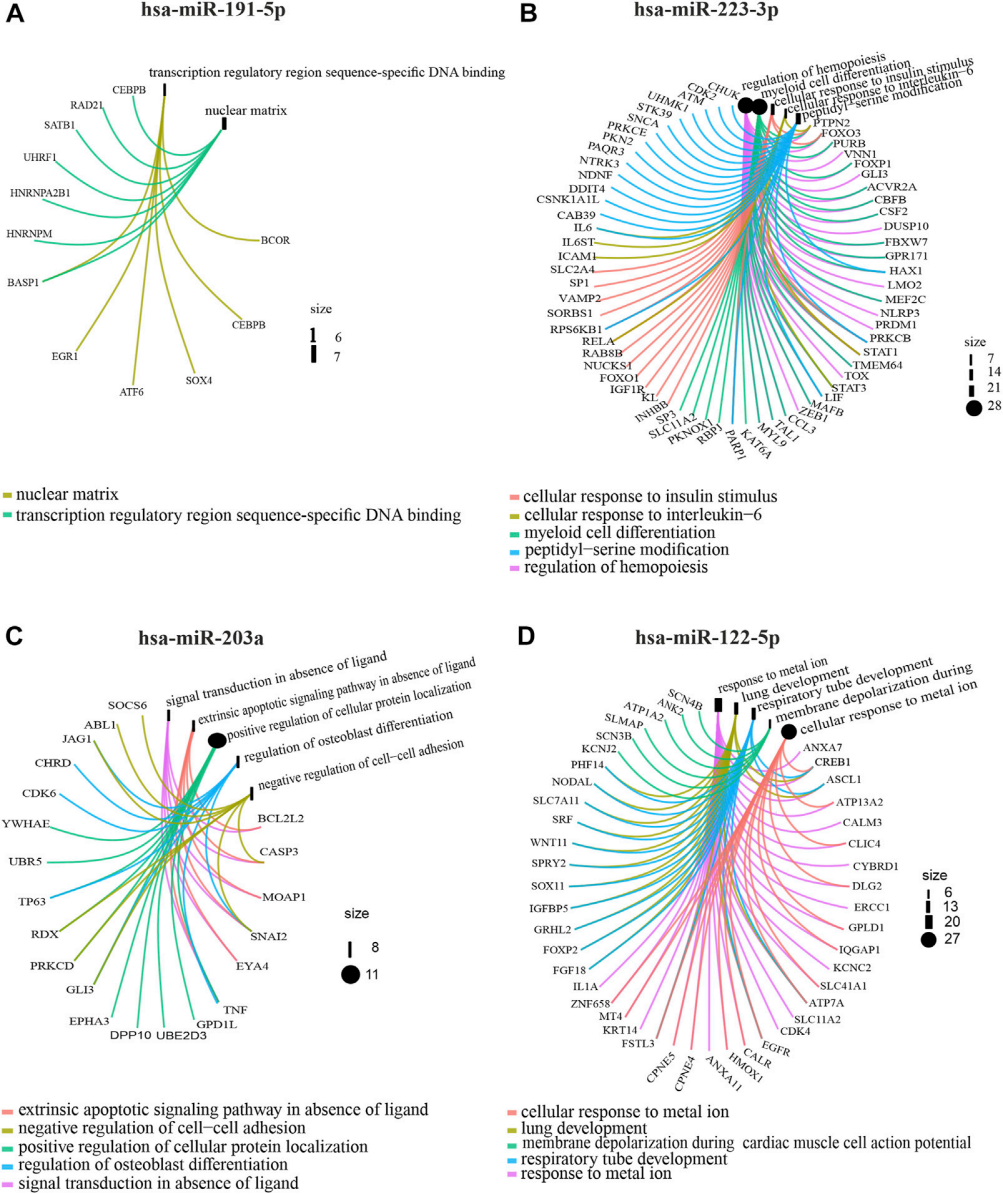
Gene Ontology analysis showing circos plots of top 5 enriched biological processes and candidate target genes regulated by exosomal miRNAs in primary UM-exos compared to metastatic UM-exos. Two most upregulated: hsa-miR-191-5p **(A)**, hsa-miR-223-3p **(B)** and 2 most downregulated: hsa-miR-203a **(C)** and hsa-miR-122-5p **(D)** miRNA are shown. Target genes involved in the particular GO terms are depicted by colored connecting lines. The size of a node corresponds to number of genes enriched in given GO term.

In order to determine the miRNA expression profiles in UM-derived exosomes, samples from 3 patients with primary (primary UM-exos) and 3 with metastatic disease (metastatic UM-exos) were subjected to high-throughput next-generation sequencing. Results revealed around 80 miRNAs to be differentially expressed (DE) in patient-derived metastatic UM-exos compared to primary UM-exos (Figure 2A). However, only 9 DE miRNAs reached statistical significance, with *p*-values ≤0.05 (Figure 2B). Hsa-miR-191-5p and hsa-miR-223-3p were downregulated, while hsa-miR-203a, hsa-miR-139-3p, hsa-miR-122-5p, hsa-miR-486-5p, hsa-miR-144-5p, hsa-miR-10b-5p and hsa-miR-483-5p were upregulated in metastatic UM-exos when compared with primary UM-exos (Figure 2C). Further, to establish their role in biological processes, we performed screening of target genes for selected differentially expressed miRNAs using the SpidermiR package, which included several databases (DIANA, Miranda, PicTar, TargetScan, miRTAR, miRwalk). Altogether, we have found 2850 target genes regulated by our miRNAs of interest, of which 700 were validated targets. Next, using the clusterProfiler package and DAVID annotation tool, we analyzed the enrichment of the validated target genes in biological processes. The results were visualized as circos plots, connecting the enriched biological processes with target genes, with a node size corresponding to the number of genes involved (Figure 3 and Supplementary Figure S1). This analysis revealed that hsa-miR-191-5p is involved in transcriptional processes and double strand breaks repair processes (Figure 3A). Amongst other functions, hsa-miR-223-3p, a miRNA that is highly expressed in UM-exos is regulating cell cycle, proliferation and apoptosis by targeting *CDK2, ATM* and *PKN2* (Figure 3B). Hsa-miR-203a in metastatic UM was shown to downregulate processes responsible for cell adhesion and apoptosis (Figure 3C). Circos plots further showed that hsa-miR-122-5p, which was profoundly upregulated in our metastatic UM samples, is implicated in stemness of cells and melanoma development by targeting Nodal growth differentiation factor (*NODAL*), *SOX11*, *WNT11* and *CDK4* (Figure 3D).

Hsa-miR-486-5p regulated Golgi vesicle transport, the WNT signaling pathway and regulation of cell-cell signaling by WNT (Supplementary Figure S1A). Melanosome transport and establishment of melanosome localization is regulated by hsa-miR-139-3p through targeting *RAB1A* and *RAB11B* genes (Supplementary Figure S1B) Hsa-miR-10b-5p was increased in metastatic UM and is connected to cell growth and homeostasis, transcription and splicing, and the regulation of escape from apoptosis by directly targeting *TP53*, *BCL6, CDK6* and *CDKN2a* (Supplementary Figure S1C) Hsa-miR-144-5p, a miRNA which is downregulated in UM-exos was significantly enriched in endothelium development, tight junction assembly and organization, and regulation of epidermis development by targeting *MET*, *SMAD4*, *ROCK1* and *ROCK2* (Supplementary Figure S1D). Finally, hsa-miR-483-5p was shown to have a role in RHO-mediated activation of serum response factor (SRF) by targeting RHO, MAPK and SRF genes (Supplementary Figure S1E). These results indicate the functional importance of the detected miRNAs for processes that may lead or enhance UM progression and metastasis.

### 3.3 Characterization of exosomal miRNAs as markers for early detection of UM

**FIGURE 4:**
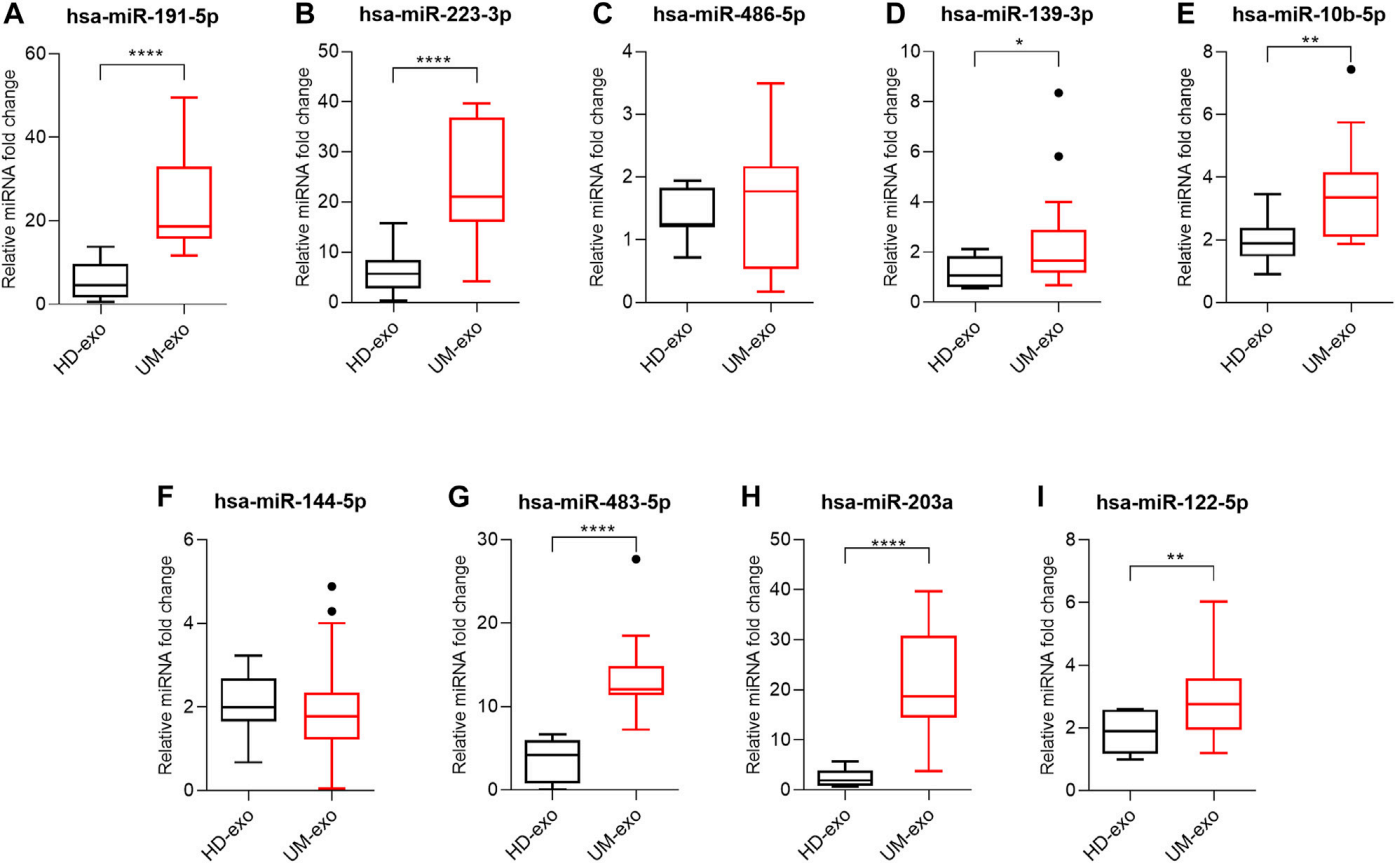
Real-time qPCR analysis of hsa-miR-191-5p **(A)**, hsa-miR-223-3p **(B)**, hsa-miR-486-5p **(C)**, hsa-miR-139-3p **(D)**, hsa-miR-10b-5p **(E)**, hsa-miR-144-5p **(F)**, hsa-miR-483-5p **(G)**, hsa-miR-203a **(H)**, hsa-miR-122-5p **(I)** in exosomes derived from healthy donors (HD-exo, *n* = 20) and patients with uveal melanoma (UM-exo, *n* = 20), regardless of the stage of disease. The results are displayed as relative miRNA fold change calculated as log2-ΔΔCt. Data were normalized to an exogenous spike-in control (hsa-miR-6351-5p mimic). The graph represents median ±SD, outliers are marked as dots. The statistical differences between the two groups were analyzed with either *t*-test with Welch correction (for samples following Gaussian distribution) or Mann-Whitney test (for samples not following Gaussian distribution), where: *p < 0.05, **p < 0.01, ***p < 0.001, ****p < 0.0001.

Next, the results of high-throughput analyses were confirmed by RT-qPCR. The validation group consisted of 40 exosome samples isolated from 20 healthy donors (HD-exo), 9 patients with primary and 11 with metastatic UM. First, we analyzed the usefulness of the selected 9 DE miRNAs in detecting UM, regardless of the disease stage. We analyzed their expression in extracellular vesicles derived from UM patients (all 20 patients’ samples, regardless of disease stage, called UM-exos) and compared expression levels with those derived from healthy donors. The analysis of hsa-miR-191-5p (Figure 4A), hsa-miR-223-3p (Figure 4B), hsa-miR-139-5p (Figure 4D), hsa-miR-10b-5p (Figure 4E), hsa-miR-483-5p (Figure 4G), hsa-miR-203a (Figure 4H) and hsa-miR-122–5 (Figure 4I) indicated significantly higher expression in UM-exos than in HD-exos. Only hsa-miR-486-5p (Figure 4C) and hsa-miR-144-5p (Figure 4F), did not reach statistical significance in differential expression.

**FIGURE 5:**
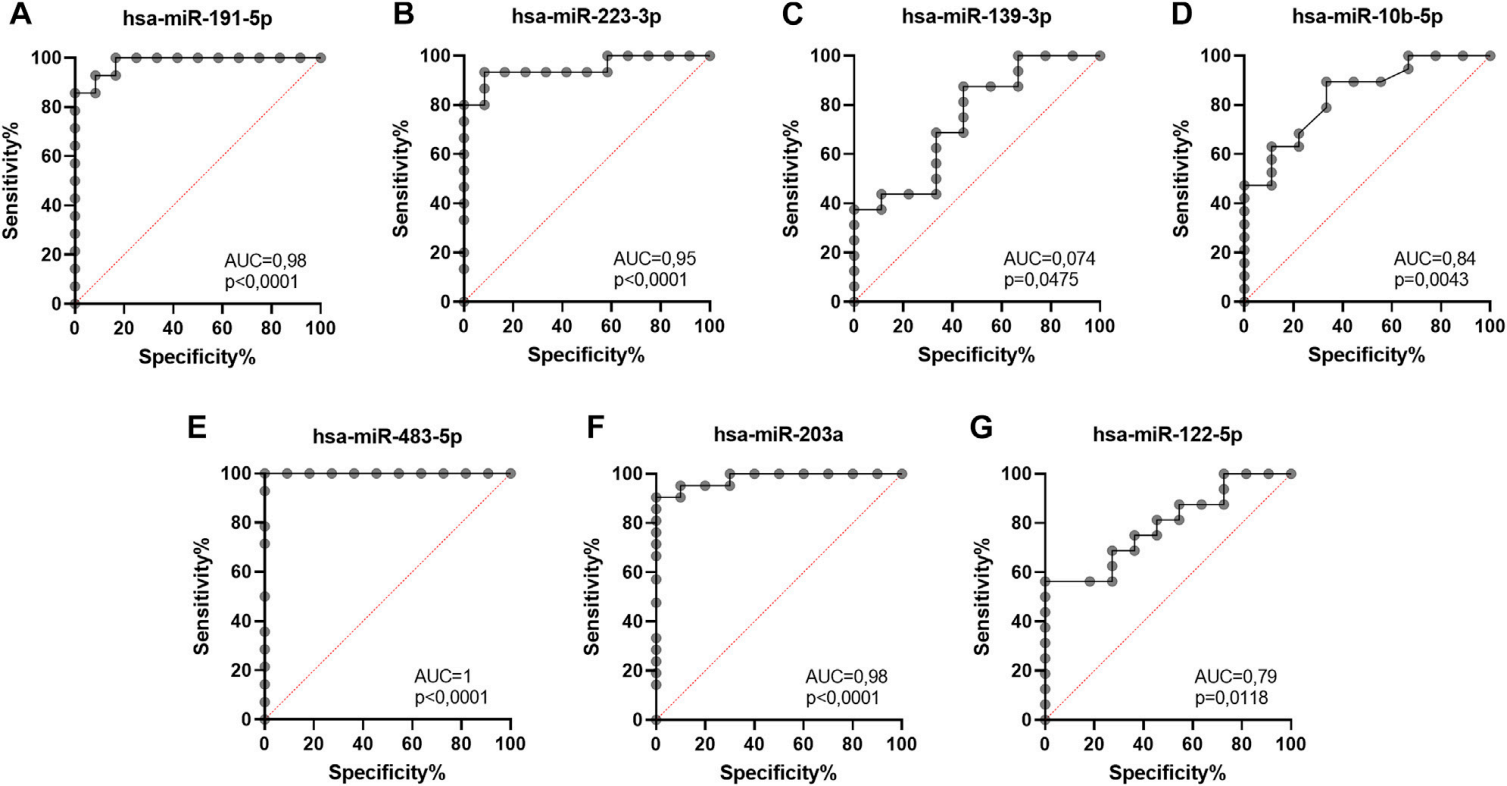
Receiver operating characteristic (ROC) curve analysis of differentially expressed hsa-miR-191-5p **(A)**, hsa-miR-223-3p **(B)**, hsa-miR-139-3p **(C)**, hsa-miR-10b-5p **(D)**, hsa-miR-483-5p **(E)**, hsa-miR-203a **(F)**, hsa-miR-122-5p **(G)** in EVs derived from heathy donors and UM patients regardless of the disease stage, based on RT-qPCR data. AUC—area under curve; p = *p* value.

To validate the diagnostic usefulness of differentially expressed miRNAs in the early detection of UM development, we performed receiver operating characteristic (ROC) curve analysis for miRNAs that reached statistical significance. We showed that the most promising candidates for diagnostic biomarkers to distinguish healthy individuals from UM patients regardless of the stage of the disease were hsa-miR-191-5p (Figure 5A), hsa-miR-223-3p (Figure 5B), hsa-miR-483-5p (Figure 5E) and hsa-miR-203a (Figure 5F). These miRNAs were characterized by the highest sensitivity (hsa-miR-483-5*p* = 100%; hsa-miR-203 = 90,5%; hsa-miR-191-5*p* = 85,7%; hsa-miR-223-3*p* = 80%) and specificity (hsa-miR-483-5*p* = 91%; hsa-miR-203 = 90%; hsa-miR-191-5*p* = 92%; hsa-miR-223-3*p* = 92%) in detecting UM development. Sensitivity and specify of hsa-miR-139-5p (Figure 5C), hsa-miR-10b-5p (Figure 5D) and hsa-miR-122–5 (Figure 5G) in discriminating between healthy individuals and UM patients did not exceed 60%.

### 3.4 Characterization of exosomal miRNAs as markers of UM metastatic spread

**FIGURE 6:**
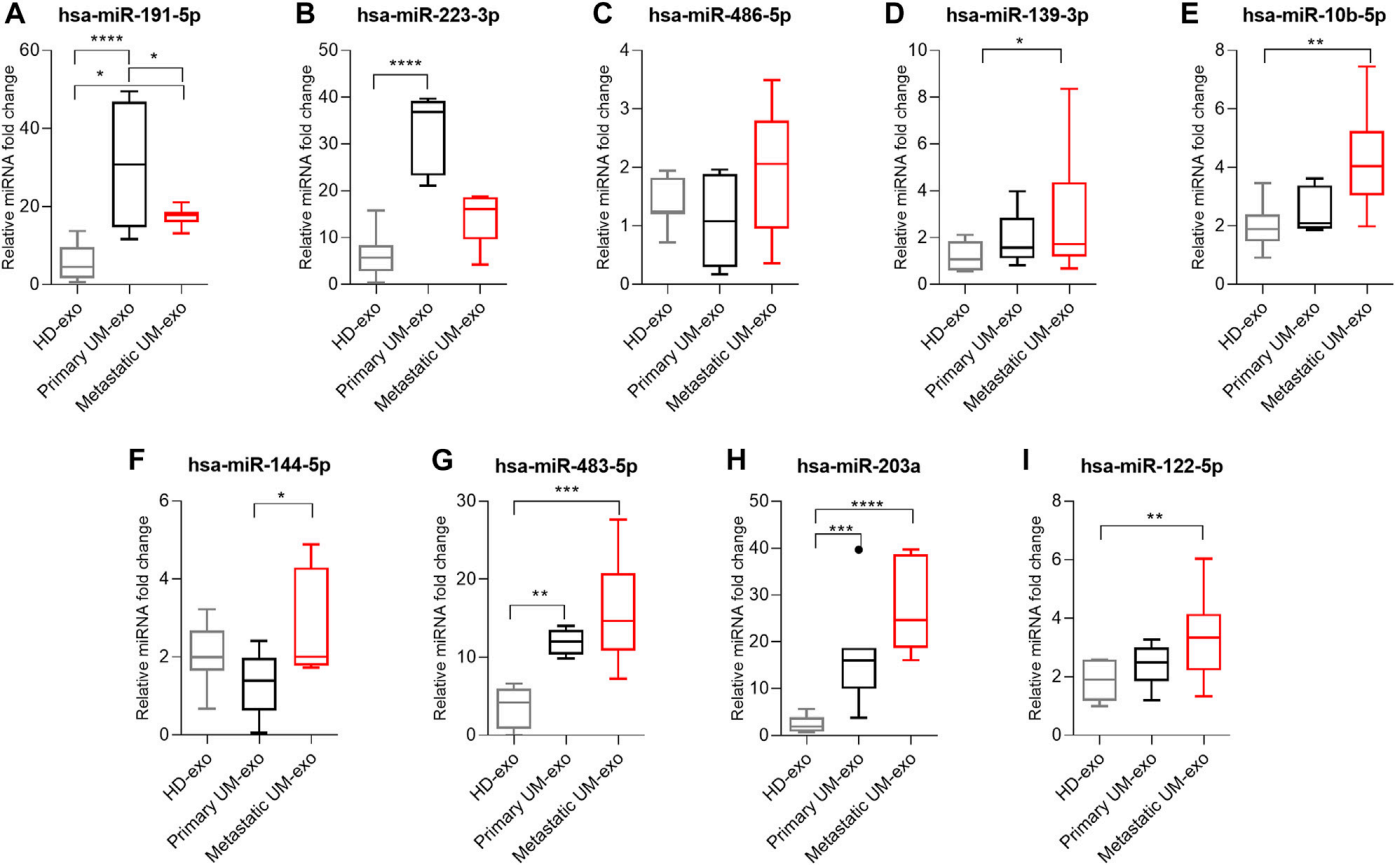
Real-time qPCR validation of hsa-miR-191-5p **(A)**, hsa-miR-223-3p **(B)**, hsa-miR-486-5p **(C)**, hsa-miR-139-3p **(D)**, hsa-miR-10b-5p **(E)**, hsa-miR-144-5p **(F)**, hsa-miR-483-5p **(G)**, hsa-miR-203a **(H)**, hsa-miR-122-5p **(I)** in exosomes derived from healthy donors (HD-exo, n=20), patients with primary (primary UM-exo, *n* = 9) and metastatic disease (Metastatic UM-exo, *n* = 11). The results are displayed as relative miRNA fold change calculated as log2-ΔΔCt. Data was normalized to exogenous spike-in: hsa-miR-6351-5p mimic. The graph represents median ±SD, outliners are marked as dots. The statistical differences between the two groups were analyzed with Two-way ANOVA or Kruskal-Wallis test with Dunn's post hoc multiple comparison test, where: *p < 0.05, **p < 0.01, ***p < 0.001, ****p < 0.0001.

To validate the high-throughput analysis, we analyzed the expression of 9 selected DE miRNAs by RT-qPCR in 3 groups: healthy donors (HD-exos), primary UM (primary UM-exos) and metastatic UM (metastatic UM-exos). Evaluation of selected miRNAs revealed that the expression of hsa-miR-191-5p (Figure 6A) and hsa-miR-223-3p (Figure 6B) was upregulated, while expression of hsa-miR-486-5p (Figure 6C), hsa-miR-139-5p (Figure 6D), miR-10b-5p (Figure 6E), hsa-miR-144-5p (Figure 6F), hsa-miR-483-5p (Figure 6G), hsa-miR-203a (Figure 6H) and hsa-miR-122–5 (Figure 6I) was downregulated in primary UM-exos compared to metastatic UM-exos, confirming results obtained from NGS analysis. However, only differences in expression of hsa-miR-191-5p (Figure 6A) and hsa-miR-144-5p (Figure 6F) reached statistical significance. We also showed that hsa-miR-203a (Figure 6B), hsa-miR-144-5p (Figure 6F), hsa-miR-10b-5p (Figure 6E), hsa-miR-483-5p (Figure 6G),, hsa-miR-139-5p (Figure 6D), and hsa-miR-122-5p (Figure 6I) were significantly upregulated in the metastatic UM-exos compared to HD-exos, while hsa-miR-223-3p (Figure 6B), hsa-miR-203a (Figure 6B) and hsa-miR-483-5p (Figure 6G) were significantly upregulated in primary UM-exos, compared to the HD-exos. Hsa-miR-144-5p (Figure 6F) was the only miRNA, whose expression was decreased in primary UM-exos compared to HD-exos, although the result did not reach statistical significance.

**FIGURE 7:**
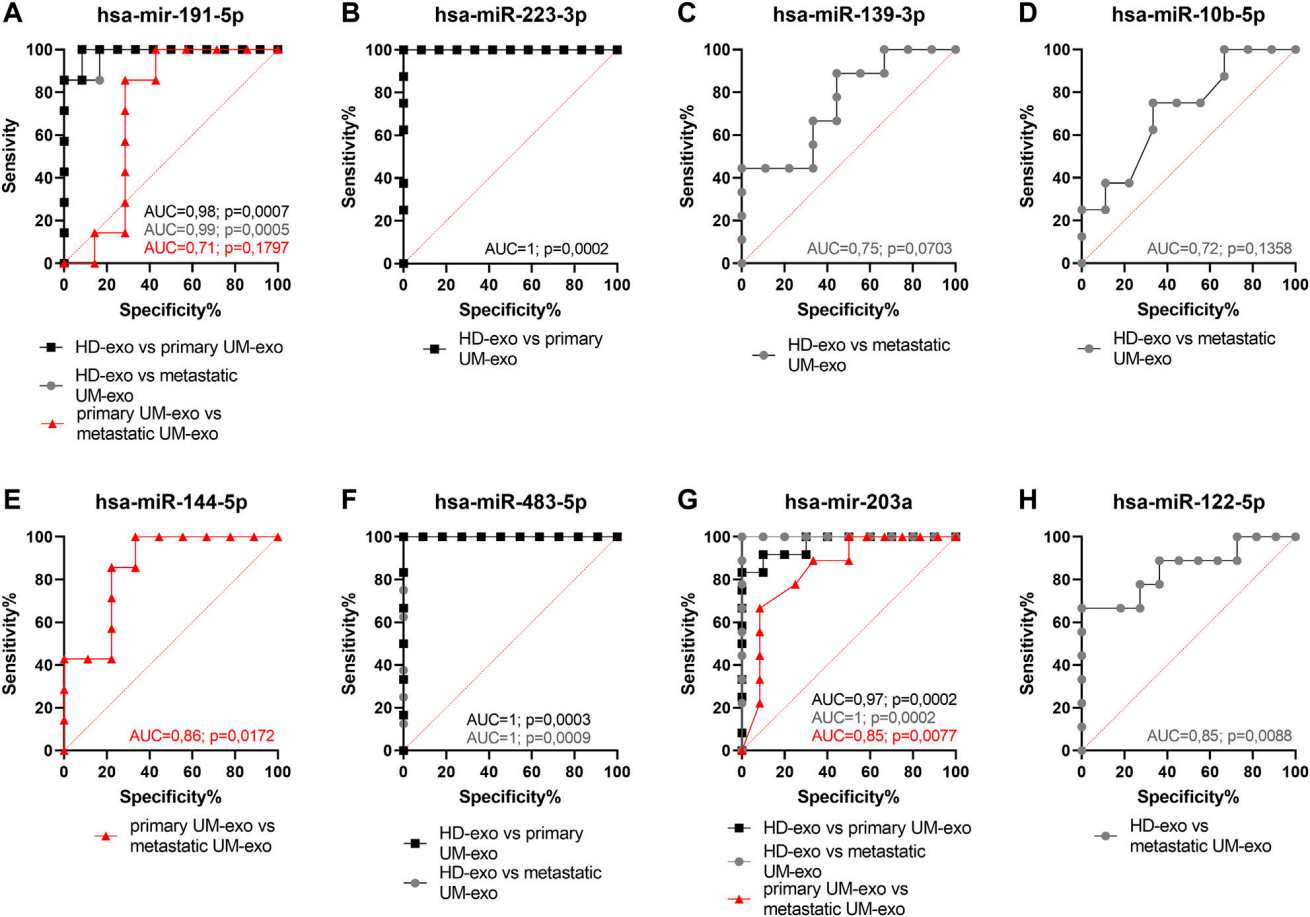
Receiver operating characteristic (ROC) curve analysis of differentially expressed hsa-miR-191-5p **(A)**, hsa-miR-223-3p **(B)**, hsa-miR-139-3p **(C)**, hsa-miR-10b-5p **(D)**, hsa-miR-144-5p **(E)**, hsa-miR-483-5p **(F)**, hsa-miR-203a **(G)**, hsa-miR-122-5p **(H)** in EVs derived from heathy donors and UM patients divided into primary and metastatic groups, based on RT-qPCR data. AUC—area under curve; p = *p* value.

To confirm the specificity and sensitivity of obtained results and their overall utility for stratification of patients prone to metastatic disease, we have performed the ROC curve analysis for each statistically significant differentially expressed miRNA, to determine their diagnostic usefulness in distinguishing between healthy individuals, primary and metastatic UM patients’ groups (Figure 7).

Hsa-miR-191-5p (Figure 7A) and hsa-miR-223-3p (Figure 7B) were highly specific (both 92%) and sensitive (100% and 85%, respectively) markers to detect early stages of UM in HD-exos vs primary UM-exo samples. Although the difference in expression between HD-exos and metastatic UM-exos of hsa-miR-139-5p (Figure 7C), hsa-miR-10b-5p (Figure 7D) and hsa-miR-122-5p (Figure 7H) reached statistical significance, those miRNAs had low sensitivity (ranging from 25 to 66%) in detecting metastatic UM. The hsa-miR-144-5p did not indicate the development of uveal melanoma, but it may be used to differentiate between primary and metastatic UM patient groups (primary UM-exos vs metastatic UM-exos). However, due to the low specificity and sensitivity, reaching only 89% and 43%, respectively, it has limited value as a single biomarker (Figure 7E). Consistent with the results from the undivided UM patient groups (HD-exos vs UM-exos), hsa-miR-483-5p was shown to be the most promising candidate to detect UM development regardless of the stage of the disease, reaching specificity and sensitivity of 91% and 100%, respectively, in distinguishing between HD-exos and primary UM-exos, and between HD-exos and metastatic UM-exos (Figure 7F). Hsa-miR-203a was characterized by similarly high specificity and sensitivity (90% and 100% respectively) in discriminating between HD-exos vs primary UM-exos samples (Figure 7G). Additionally, hsa-miR-191-5p (Figure 7A) and hsa-miR-203a (Figure 7G) may be used to distinguish between HD-exos and metastatic UM-exos or primary UM-exos and metastatic UM-exos.

Taken together, we show that two down-regulated miRNAs (hsa-miR-483-5p and hsa-miR-203a) and two up-regulated miRNAs (hsa-miR-191-5p and hsa-miR-223-3p) extracted from serum-derived extracellular vesicles are promising biomarker candidates for early detection of uveal melanoma development (Table 2). Moreover, hsa-miR-144-5p and hsa-miR-191-5p have the potential to distinguish between patients with primary and metastatic UM. However, due to relatively low specificity and sensitivity, these markers should be used in combination with other indicators supported by clinical examination and observation.

**TABLE 2:** Summary of differentially expressed miRNAs of interest utility as prognostic marker for UM development and progression, based on results obtained from qPCR and ROC analyzes. ↑- increased expression; ↓ - decreased expression.

	Possibility to utilize as prognostic marker			
	Development of UM regardless of disease stage (HD-exos vs UM-exos)	Primary UM development (HD-exos vs primary UM-exos)	Metastatic UM development (HD-exos vs metastatic UM-exos)	UM progression (primary UM-exos vs metastatic UM-exos)
miRNA				
hsa-miR-191-5p	Yes (↑ in UM-exos)	Yes (↑ in primary UM-exos)	Yes (↑ in metastatic UM-exos)	Yes (↓ in metastatic UM-exos)
hsa-miR-223-3p	Yes (↑ in UM-exos)	Yes (↑ in primary UM-exos)	No	No
hsa-miR-486-5p	No	No	No	No
hsa-miR-139-3p	Yes (↑ in UM-exos)	No	Yes (↑ in metastatic UM-exos)	No
hsa-miR-10b-5p	Yes (↑ in UM-exos)	No	Yes (↑ in metastatic UM-exos)	No
hsa-miR-144-5p	No	No	No	Yes (↑ in metastatic UM-exos)
hsa-miR-483-5p	Yes (↑ in UM-exos)	Yes (↑ in primary UM-exos)	Yes (↑ in metastatic UM-exos)	No
hsa-miR-203a	Yes (↑ in UM-exos)	Yes (↑ in primary UM-exos)	Yes (↑ in metastatic UM-exos)	No
hsa-miR-122-5p	Yes (↑ in UM-exos)	No	Yes (↑ in metastatic UM-exos)	No

## 4 Discussion

Exosomes are known to function as a repository for molecules reflecting parental cell type. It has been assessed that their amounts and compositions correspond to early onset of several diseases, also indicating disease stages (Kalluri and LeBleu, 2020). The existence of UM-specific exosomes has already been confirmed *in vivo* in the vitreous humor, blood serum and the hepatic circulation system (Eldh et al., 2014; Ragusa et al., 2015; Luz Pessuti et al., 2022) (Surman et al., 2019; Wróblewska et al., 2021) and *in vitro* in cell culture supernatants (Tsering et al., 2020; Ambrosini et al., 2022). In earlier studies, we have focused on analyzing the molecular cargo of UM-derived exosomes’ protein or general nucleic acids content. In this study, we present the first comprehensive analysis and comparison of exosome-derived miRNAs from serum samples of UM patients with primary and metastatic tumors compared to healthy donors, focusing on their potential use as biomarkers for early detection of UM development and progression into metastatic disease.

In our study, we have focused on miRNAs differentially expressed between primary and metastatic UM exosomes and showed that detected miRNAs are involved in several cellular processes responsible for cancer progressions, such as cell adhesion, homeostasis, melanosome formation and transport, insulin signaling, WNT and IL-6 signaling. Moreover, the herein detected exosomal miRNAs are also known to modulate immune cells’ activity, including NK cells (hsa-miR-93, hsa-miR-20a, hsa-miR-10b, hsa-miR-155, hsa-miR-223), monocytes and macrophages (hsa-miR-23a, hsa-miR-27a) and T cells (hsa-miR-23a, hsa-miR-27a) (Omar et al., 2019).

Several studies have analyzed miRNA expression in sera of melanoma patients and showed their potential as prognostic markers. Stark et al. presented a panel of six miRNAs (hsa-miR-16, hsa-miR-145, hsa-miR-146a, hsa-miR-204, hsa-miR-211, and hsa-miR-363-3p) that could distinguish between patients with UM and uveal nevi, with hsa-miR-211 being able to distinguish between primary and metastatic tumors accurately additionally (Stark et al., 2019). Achberger et al., reported plasma levels of hsa-miR-20a, hsa-miR-125b, hsa-miR-146a, hsa-miR-155, hsa-miR-181a, and hsa-miR-233 to be elevated in UM patients compared to healthy controls and further increasing during metastatic spread (Achberger et al., 2014). Hsa-miR-146a was also reported to be increased in sera of UM patients, but no correlation with clinical data was detected (Russo et al., 2016).

To the best of our knowledge, only two studies have investigated exosomal miRNA cargo in uveal melanoma. Eldh et al. analyzed miRNA profile in exosomes obtained from the liver perfusate of metastatic uveal melanoma patients and compared it with exosomes derived from mast cells, melanoma and lung, and breast cancer cell lines. They confirmed the presence of uveal melanoma-specific exosomes in hepatic circulation and determined the expression of miRNAs (hsa-miR-370, hsa-miR-210, hsa-miR-320a, hsa-miR-124, hsa-miR-107 and hsa-miR-486-5p) typical for patients’ samples and melanoma cell lines, involved in the regulation of cancer-related pathways (melanoma, prostate and glioma), hedgehog signaling, insulin signaling and focal adhesion, similar to our findings (Eldh et al., 2014). Also, Ragusa et al. analyzed miRNA profiles in the vitreous humor and vitreal exosomes from sera of UM patients of unknown disease stages by microarray. They showed that hsa-miR-146a was significantly upregulated in exosomes derived from the vitreous humor of UM patients compared to healthy controls. Based on these results, exosome-derived hsa-miR-146a might be a potential biomarker for uveal melanoma detection (Ragusa et al., 2015). Although we have also detected high levels of hsa-mir-146a in UM-derived exosomes, we did not see significant differences between primary and metastatic UM. The utility of oncogenic hsa-miR-146a as a marker for UM progression would therefore need to be confirmed in a larger cohort.

Recent studies reported the loss of hsa-miR-144 expression in UM cells. Sun et al. showed that overexpression of hsa-miR-144 inhibits UM cells proliferation and migration by down-regulating c-Met (Sun et al., 2015). Similar results were reported by Amaro and Croce et al. who showed that hsa-miR-144 and hsa-miR-122 are tumor suppressing miRNAs, whose loss of expression leads to increased cell proliferation, migration and reduced apoptosis by directly targeting pro-invasive ADAM10 and c-Met molecules in UM (Amaro et al., 2020). Moreover, it was shown that hsa-miR-144-5p may mediate metastatic spread of UM, due to its involvement in the regulation of genes responsible for loss of cell adhesion and cell-to-cell connections (Qu et al., 2013; Zhou et al., 2021). In our study, we also detected lower expression of hsa-miR-144 in primary UM EVs compared to the healthy donor group. Surprisingly, the expression in metastatic UM EVs was significantly increased. Similarly, the expression of hsa-miR-122 and hsa-miR-10b-5p was also increased in UM-*versus* HD-derived exosomes, but it was related to its significant upregulation in metastatic patients. In case of miR-122-5p, there are several data, which indicate its correlation with the development of metastatic disease in few malignancies, affecting the metabolism and proliferation of cells (Fong et al., 2015; Heinemann et al., 2018; Wang et al., 2019). The other one, hsa-miR-10b-5p, is responsible for the downregulation of *BAP1* in metastatic UM, whose loss is highly correlated with progression and worse outcomes of UM (Robertson et al., 2017). One possible explanation for this discrepancy is the difference in input material - Amaro and Croce et al. analyzed miRNA expression in tumor samples and cell lines only. It has been shown that miRNA levels are often higher in EVs than in parental cells, as some miRNAs are preferentially loaded into EVs (Ohshima et al., 2010; Goldie et al., 2014). However, further studies on this subject are required to explain this phenomenon fully.

Triozzi et al. reported elevated levels of hsa-miR-223 in plasma of UM patients with monosomy 3 compared to disomy 3. Also, the level of this miRNA was higher in UM patients, regardless of chromosome 3 status, when compared with healthy donor plasma (Triozzi et al., 2016). Joshi et al. showed that UM cell lines express and secrete hsa-miR-146a, hsa-miR-155 and hsa-miR-223, which are responsible for decreased activity of NK cells and reduced UM sensitivity to NK-mediated cytolysis (Joshi et al., 2016). Similar to these cell line-derived data, we also show augmented expression of both hsa-miR-146a, hsa-miR-155 and hsa-miR-223 in UM exosomes (compared to HD samples), but without significant differences between primary and metastatic patients.

Early detection of uveal melanoma development and progression into metastatic disease remains a challenge since no reliable, easy-to-use molecular biomarkers indicate the presence and/or disease progression. Previously, we have reported potential protein biomarkers derived from UM extracellular vesicles that allow the distinguishment of healthy individuals and uveal melanoma patients with either primary or metastatic disease. We also showed the possibility of using these markers to detect the metastatic spread of UM (Wróblewska et al., 2021). Our current findings confirmed several miRNAs previously reported to be differentially expressed in UM patients’ serum/EVs samples (hsa-miR-144-5p, hsa -miR-223-3p, hsa -miR-203a, hsa -miR-483 and hsa -miR-486-5p). We also report extracellular vesicles-derived hsa -miR-191-5p as a novel biomarker for early detection of UM. In addition to this, we have identified a set of miRNAs specifically distinguishing primary from metastatic UM (hsa-miR-144-5p, hsa-miR-203a and hsa-miR-191-5p), and these miRNAs are therefore applicable candidates for UM disease management to be explored in a larger cohort. Although our results require confirmation in a larger cohort of UM patients, we believe that the described molecules have the potential to be utilized as prognostic factors for determining risk and early detection of disease progression.

## Data Availability

The datasets presented in this study can be found in online repositories. The names of the repository/repositories and accession number(s) can be found below: https://www.ncbi.nlm.nih.gov/, PRJNA864071.
