# Supplementary figures and images for: MiRNAs from serum-derived extracellular vesicles as biomarkers for uveal melanoma progression
Source: Front Cell Dev Biol. 2022 Dec 22;10:1008901. doi: 10.3389/fcell.2022.1008901 (PMC9814164; doi:10.3389/fcell.2022.1008901)

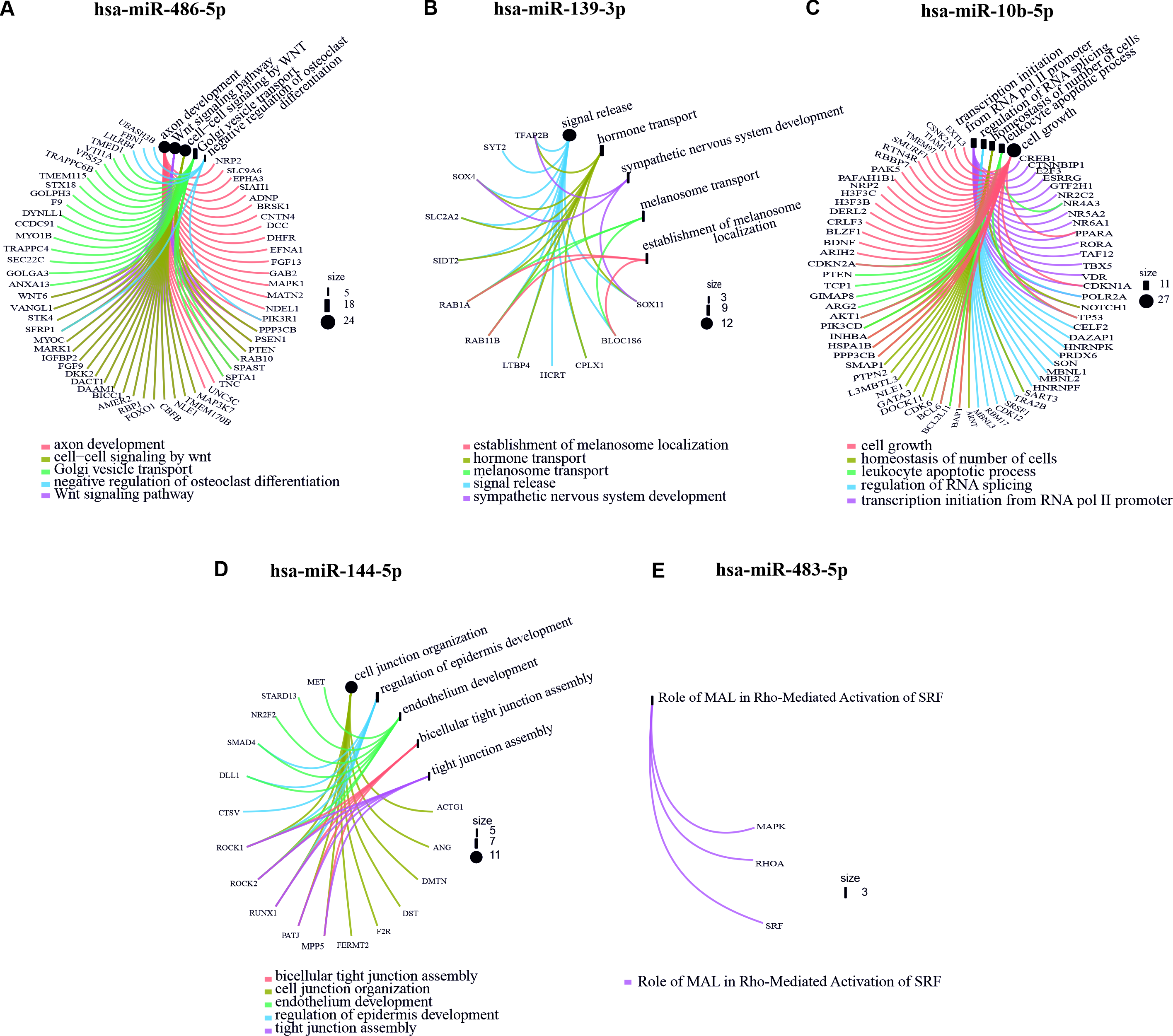

Supplement: Supplementary file 1 [file Image1.TIF]
