# Supplementary material for: MiRNAs from serum-derived extracellular vesicles as biomarkers for uveal melanoma progression
Source: Front Cell Dev Biol. 2022 Dec 22;10:1008901. doi: 10.3389/fcell.2022.1008901 (PMC9814164; doi:10.3389/fcell.2022.1008901)
Supplement: Supplementary file 2 [file DataSheet1.docx]

Supplementary Material

**Supplementary Figure 1.** Gene Ontology analysis showing top 5 enriched biological processes and candidate target genes regulated by exosomal miRNAs downregulated in primary UM-exos comparing to metastic UM-exos. Target genes involved in the particular GO terms are depicted by colored connecting lines. The size of a node corresponds to number of genes enriched in given GO term.
